# Massively parallel sequencing fails to detect minor resistant subclones in tissue samples prior to tyrosine kinase inhibitor therapy

**DOI:** 10.1186/s12885-015-1311-0

**Published:** 2015-04-15

**Authors:** Carina Heydt, Niklas Kumm, Jana Fassunke, Helen Künstlinger, Michaela Angelika Ihle, Andreas Scheel, Hans-Ulrich Schildhaus, Florian Haller, Reinhard Büttner, Margarete Odenthal, Eva Wardelmann, Sabine Merkelbach-Bruse

**Affiliations:** 1 Institute of Pathology, University Hospital Cologne, Kerpener Str. 62, 50937 Cologne, Germany; 2 Institute of Pathology, University Hospital Erlangen, Krankenhausstraße 8-10, 91054 Erlangen, Germany; 3 Institute of Pathology, University Hospital Göttingen, Robert-Koch-Strasse 40, 37075 Göttingen, Germany; 4 Gerhard-Domagk-Institute of Pathology, University Hospital Münster, Albert-Schweitzer-Campus 1, Gebäude D17, 48149 Münster, Germany

**Keywords:** NGS, Parallel sequencing, Sensitive methods, GIST, Pre-existing, Minor subclone, Low frequency mutation, Resistance

## Abstract

**Background:**

Personalised medicine and targeted therapy have revolutionised cancer treatment. However, most patients develop drug resistance and relapse after showing an initial treatment response. Two theories have been postulated; either secondary resistance mutations develop de novo during therapy by mutagenesis or they are present in minor subclones prior to therapy. In this study, these two theories were evaluated in gastrointestinal stromal tumours (GISTs) where most patients develop secondary resistance mutations in the *KIT* gene during therapy with tyrosine kinase inhibitors.

**Methods:**

We used a cohort of 33 formalin-fixed, paraffin embedded (FFPE) primary GISTs and their corresponding recurrent tumours with known mutational status. The primary tumours were analysed for the secondary mutations of the recurrences, which had been identified previously. The primary tumours were resected prior to tyrosine kinase inhibitor therapy. Three ultrasensitive, massively parallel sequencing approaches on the GS Junior (Roche, Mannheim, Germany) and the MiSeq^TM^ (Illumina, San Diego, CA, USA) were applied. Additionally, nine fresh-frozen samples resected prior to therapy were analysed for the most common secondary resistance mutations.

**Results:**

With a sensitivity level of down to 0.02%, no pre-existing resistant subclones with secondary *KIT* mutations were detected in primary GISTs. The sensitivity level varied for individual secondary mutations and was limited by sequencing artefacts on both systems. Artificial T > C substitutions at the position of the exon 13 p.V654A mutation, in particular, led to a lower sensitivity, independent from the source of the material. Fresh-frozen samples showed the same range of artificially mutated allele frequencies as the FFPE material.

**Conclusions:**

Although we achieved a sufficiently high level of sensitivity, neither in the primary FFPE nor in the fresh-frozen GISTs we were able to detect pre-existing resistant subclones of the corresponding known secondary resistance mutations of the recurrent tumours. This supports the theory that secondary *KIT* resistance mutations develop under treatment by “de novo” mutagenesis. Alternatively, the detection limit of two mutated clones in 10,000 wild-type clones might not have been high enough or heterogeneous tissue samples, per se, might not be suitable for the detection of very small subpopulations of mutated cells.

**Electronic supplementary material:**

The online version of this article (doi:10.1186/s12885-015-1311-0) contains supplementary material, which is available to authorized users.

## Background

In recent years, personalised cancer medicine and the development of receptor tyrosine kinase inhibitors as well as monoclonal antibodies for targeted therapies led to dramatic improvements in cancer treatment and patient care. Nonetheless, most patients develop drug resistance and relapse after an initial treatment response [1,2]. Numerous studies have investigated the underlying mechanisms of drug resistance and showed, among others facts, that secondary mutations of the gene encoding the target protein are responsible for drug resistance [3,4]. The emergence of secondary gene mutations in a heterogeneous tumour population follows the Darwinian law. Thus far, it is not entirely understood if these mutations develop by means of mutagenesis during therapy or if secondary gene mutations are present in pre-existing minor subclones in a tumour subpopulation and are selected for during therapy [5,6]. Sensitive methods as well as mathematical models, like the Luria-Delbrück model, led to the identification of pre-existing resistant subclones prior to therapy in some tumour entities: In non-small cell lung cancer the *EGFR* resistance mutation p.T790M and in colorectal carcinoma secondary *KRAS* mutations down to a frequency of 0.01% [7,8]. In this study, primary and secondary gastrointestinal stromal tumours (GISTs) were analysed. 75 – 80% of GISTs are characterised by activating mutations in the *KIT* gene [9]. Primary unresectable or metastatic KIT positive GISTs are commonly treated with the receptor tyrosine kinase inhibitor imatinib (Glivec®, Novartis Pharma). After an initial treatment response, nearly half of the patients show tumour progression within two years [10,11]. The most common resistance mechanism is the acquisition of secondary resistance mutations in the *KIT* gene [11,12]. It is still unknown whether the secondary resistance mutations pre-exist in minor subclones or develop “de novo” during therapy [5,11,13-15]. This study investigated, using the currently available ultrasensitive methods, if secondary *KIT* mutations pre-exist in minor subclones in GISTs. For this approach, three massively parallel sequencing assays were used on the GS Junior (Roche, Mannheim, Germany) and on the MiSeq™ (Illumina, San Diego, CA, USA). The detection of pre-existing resistant subclones would be a crucial contribution to the choice of treatment course. Primary and secondary *KIT* mutations could be targeted simultaneously by a combination of tyrosine kinase inhibitors. Thus, tumour growth and progression due to resistances could be prevented.

## Methods

### Cases and immunohistochemistry

33 cases of corresponding primary and secondary formalin-fixed and paraffin embedded (FFPE) GISTs with known mutational status were selected retrospectively from the GIST and Sarcoma Registry Cologne/Bonn (Table 1). FFPE tissue samples were obtained as part of routine clinical care under approved ethical protocols complied with the Ethics Committee of the Medical Faculty of the University of Cologne, Germany and informed consent from each patient. Histological specimens were evaluated by board certified senior pathologists specialised in soft tissue pathology (E. W., H.-U. S. or R. B.). The diagnosis was based on morphology and immunohistochemistry against CD117, CD34, BCL2 (all Dako) and DOG1 (Spring Bioscience) as described previously [11,16]. The mutational status of all samples was routinely analysed by Sanger sequencing and high resolution melting analysis as described previously [5,16,17] (Table 1). Two cases (case 13 and 31) showed a high polyclonal evolution of multiple secondary *KIT* mutations.

**Table 1:** **Clinical and pathological data and mutational status of 33 primary GISTs with known recurrent lesions**

Primary tumour										Recurrent lesion	
Case No.	CD 117	CD 34	BCL2	DOG1	Tumour cell type	Sex	Age	Localisation	Primary mutation	Case No.	Secondary mutation
1	+	+	+	NA	Spindle	M	80	EGIST	11: p.W557_V559delinsF	1 a	13: p.V654A
2	+	+	-	NA	Spindle	F	59	Small intestine	11: p.W557_E561del	2 a	13: p.V654A
										2 b	17: Y823D
										2 c	13: p.V654A
3	+	(+)	(+)	NA	Mixed	M	45	Stomach	11: p.K550_V555delinsL	3 a	17: p.D820Y
4	+	+	+	NA	Spindle	F	44	Peritoneum	11: p.W557_V560delinsC	4 a	13: p.V654A
										4 b	13: p.V654A
											17: p.D820E
5	+	+	+	NA	Epitheloid	F	66	Peritoneum	11: p.V559A	5 a	13: p.V654A
6	+	+	+	NA	Spindle	F	28	Small intestine	11: N567_L576delinsI	6 a	17: p.D820G
7	+	(+)	(+)	NA	Spindle	M	66	Peritoneum	11: p.Q556_W557del	7 a	13: p.V654A
8	+	+	+	+	Spindle	F	41	Stomach	11: p.[V560G(;)N566D]	8 a	13: p.V654A
9	+	+	+	+	Mixed	M	44	Stomach	11: p.K550_K558del	9 a	14: p.T670I
10	+	NA	NA	+	Spindle	M	67	NA	11: p.V559G	10 a	17: p.D820Y
11	+	+	+	+	Spindle	F	65	Stomach	11: p.V559D	11 a	17: p.N822Y
										11 b	17: p.D820E
12	+	+		+	Spindle	M	63	Stomach	11: p.W557_K558del	12 a	13: p.V654A
13	+	-	+	+	Spindle	F	71	Small intestine	11: p.V559G	13 a	11: p.[V559G;Y578C]; [V559G;D579del]
											13: p.V654A
										13 b	11: p.[V559G]; [V559G;Y578C]
											13: p.V654A
											14: p.N680K
14	+	+	NA	NA	Mixed	F	43	Small intestine	11: p.N567_Y578delinsSCV	14 a	17: p.N822K
15	+	-	-	NA	Mixed	F	59	EGIST	11: p.D579_H580insQQLPYD	15 a	17: p.D820E
16	+	+	+	+	Mixed	M	50	Small intestine	9: p.A502_Y503dup	16 a	17: p.N822Y
17	+	-	-	+	Epitheloid	F	71	Small intestine	11: p.I563_P573del	17 a	17: p.K818_D820delinsN
18	+	+	+	+	Spindle	F	52	Stomach	11: p.K558_V560del	18 a	17: p.D820Y
19	+	+	-	+	Spindle	M	65	Stomach	11: p.W557_V560delinsC	19 a	17: p.Y823D
20	+	+	-	+	Spindle	M	42	NA	11: p.M552_V559del	20 a	14: p.T670E
21	+	+	+	+	Spindle	F	59	Stomach	11: p.W557_V559delinsC	21 a	13: p.V654A
22	+	+	-	+	Spindle	M	46	Rectum	11: p.K558_V560delinsS	22 a	13: p.V654A
23	+	+	+	+	Mixed	M	53	Stomach	11: p.V559G	23 a	13: p.V654A
24	+	+	+	+	Spindle	M	60	Stomach	11: p.W557_V559delinsF	24 a	17: p.N822K
25	+	+	(+)	NA	Mixed	M	65	Stomach	11: c.1648-5_1672del	25 a	-
26	+	-	+	NA	Epitheloid	F	75	EGIST	11: p.Y570_L576del	26 a	-
27	+	-	+	NA	Mixed	M	72	Small intestine	9: p.A502_Y503dup	27 a	-
28	+	+	-	NA	Spindle	F	42	NA	9: p.A502_Y503dup	28 a	n.n.
29	+	+	+	NA	Mixed	M	66	EGIST	11: p.M552_K558del	29 a	-
30	+	+	+	NA	Spindle	F	43	Peritoneum	11: p.W557R	30 a	13: p.K642E
											13: p.V654A
31	+	-	+	+	Epitheloid	F	64	Peritoneum	11: p.W557G	31 a	13: p.V654A
											11: p.[W557G(;) V569_Y578del]
											17: p.N822Y
32	+	+	+	+	Spindle	F	77	Stomach	11: p.W557R	32 a	17: p.N822K
33	+	+	+	+	Mixed	M	68	Small intestine	11: p.I563_P577delinsN	33 a	17: p.D820G

NA: Not known. +: Positive staining; (+): Focal positive staining. M: Male; W: Female. n.n.: Not evaluable. EGIST: Extragastrointestinal stromal tumour; a, b, c: Count of recurrent lesions of one case.

From the 33 cases, the tumour regions of five cases were divided into a total of 52 subregions of about the same size. The subregions defined for this study were selected after re-examination of their immunohistochemical staining pattern by a board certified senior pathologist (E. W.).

Additionally, nine fresh-frozen GISTs (seven primary GISTs and two metastases) with known mutational status were selected from the registry of the Institute of Pathology, University Hospital Erlangen (Table 2). All nine samples had been collected prior to therapy.

**Table 2:** **Nine fresh-frozen GISTs before therapy with known status of primary** ***KIT*** **mutation**

Case No.	Mutation primary tumour	Mutation metastasis
F1	11: p.V559G	n.a.
F2	Wt	n.a.
F3	9: p.A502_Y503dup	n.a.
F4	Wt	n.a.
F5	n.a.	11: p.V559D
F6	n.a.	Wt
F7	Wt	n.a.
F8	p.W557_V559delinsF	n.a.
F9	p.V559A	n.a.

n.a.: Not analysed. Wt: Wild-type.

### Quantitative immunohistochemistry

Quantitative immunohistochemistry was performed by whole-slide scanning with a resolution of 0.22 μm/pixel (Pannoramic 250, 3DHistech, Budapest, Hungary) and analysed with ImageJ [18]. For each subregion, three fields-of-view were analysed at a 200x magnification covering 1.85 mm^2^ and >500 tumour cells. Staining intensity was calculated by colour deconvolution.

### DNA Extraction

**Figure 1:**
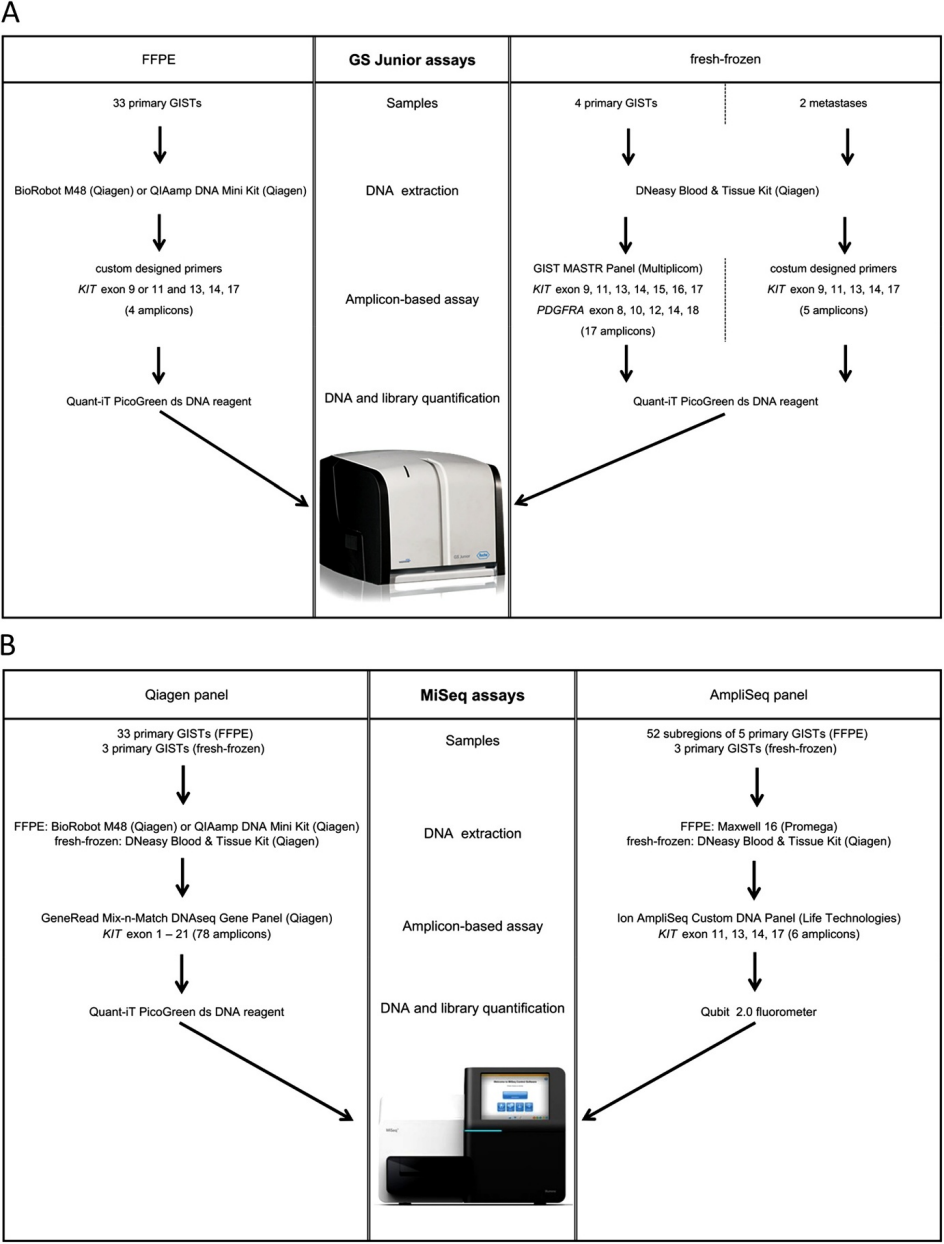
Visual depiction of the different experiments and workflows performed on the GS Junior (Roche) **(A)** and the MiSeq™ (Illumina) **(B)** with FFPE and fresh-frozen material.

Six sections of 10 μm thickness were cut from FFPE tissue blocks. After deparaffinisation, tumour areas were macrodissected from unstained slides. The tumour area was marked on a haematoxylin-eosin (H&E) stained slide by a senior pathologist (E. W., H.-U. S.). DNA was extracted with the MagAttract® DNA Mini M48 Kit (Qiagen, Hilden, Germany) on the BioRobot® M48 (Qiagen). Samples collected before the year 2010 were extracted manually with the QIAamp® DNA Mini Kit (Qiagen). DNA extraction of the subregions were performed with the Maxwell® 16 FFPE Plus Tissue LEV DNA Purification Kit (Promega, Mannheim, Germany) on the Maxwell® 16 (Promega). Fresh-frozen tissues were extracted with the DNeasy® Blood & Tissue Kit (Qiagen) (Figure 1). All extraction procedures were performed following the manufacturers’ instructions.

## Amplicon-based massively parallel sequencing

### DNA quantification

For the sensitive analysis of the primary GISTs, two massively parallel sequencing platforms were used: the GS Junior (Roche, Mannheim, Germany) and the MiSeq™ (Illumina, San Diego, CA, USA). All samples were quantified in duplicates by the Quant-iT™ dsDNA HS Assay (Life Technologies, Darmstadt, Germany) on the Qubit® 2.0 fluorometer (Life Technologies) and with the Quant-iT™ PicoGreen® dsDNA reagent (Life Technologies) (Figure 1).

### GS Junior (Roche)

For the analysis of FFPE samples on the GS Junior (Roche), a custom designed library was prepared according to the Roche guidelines covering *KIT* exon 13, 14 and 17 combined with either exon 9 or 11 (Figure 1). Target specific primers are listed in Additional file 1. 100 – 150 ng of genomic DNA were used for library preparation.

For library preparation of the fresh-frozen primary GISTs, 75 ng DNA were amplified using custom designed primers (Additional file 2) and Phusion Hot Start Flex DNA Polymerase (New England Biolabs, Ipswich, MA, USA) according to manufacturer’s instructions.

For the fresh-frozen metastases the GIST MASTR (Multiplicom, Niel, Belgium) and the 454 MID kit 1–8 (Multiplicom) were used according to manufacturer’s instructions (Figure 1).

Libraries were purified, quantified and diluted to a final concentration of 1 x 10^6^ molecules. 10 – 14 samples were multiplexed, clonally amplified by emulsion PCR and sequenced on the GS Junior (Roche) following manufacturer’s instructions.

### MiSeq™ (Illumina)

Two amplicon-based assays were used on the MiSeq™ (Illumina): a GeneRead Mix-n-Match DNAseq Gene Panel (Qiagen panel, Qiagen) for the whole *KIT* gene consisting of 78 amplicons and an Ion AmpliSeq™ Custom DNA Panel (AmpliSeq panel, Life Technologies) for exon 11, 13, 14 and 17 of the *KIT* gene with six amplicons (Additional file 3). All 33 primary GISTs were evaluated with the Qiagen panel. The 52 subregions of the five subdivided cases were analysed with the AmpliSeq panel. Three fresh-frozen samples were investigated with both assays (Figure 1).

Analysis with the Qiagen panel was performed according to the GeneRead DNAseq Gene Panel Handbook (Qiagen). With the AmpliSeq panel, 10 ng of DNA were amplified as described previously [19]. In brief, barcodes were ligated to multiplex PCR products and targets were enriched with the Ion AmpliSeq™ Library Kit 2.0 (Life Technologies).

All samples were quantified and diluted. 5 – 6 (Qiagen panel) or 15 – 24 samples (AmpliSeq panel) were multiplexed and sequenced on the MiSeq™ (Illumina) following manufacturer’s instructions.

### Bioinformatics

GS Junior (Roche) sequencing reads were aligned to the human reference genome 19 (hg19) and analysed with the Amplicon Variant Analyser (AVA, Roche).

FASTQ files were generated and exported on the MiSeq™ (Illumina). The FASTQ files were aligned to the reference genome (NCBI build 37/hg19) using BWA and BLAT algorithms. Variants were called with an in-house pipeline developed by Peifer et al., which is based on the general cancer genome analysis pipeline [20]. Mapped reads and called variants were combined in a BAM file and data were visualised with the Integrative Genomics Viewer (IGV) [21].

### Analysis of assay sensitivity, specificity and limit of detection

All assays were validated with a set of samples with a known mutational status. For the MiSeq™ (Illumina) assays, all 36 secondary samples and for the GS Junior a different set of 18 samples were used. The sensitivity and specificity were determined for each assay. The sensitivity is defined as the proportion of correctly identified positive events (True positive rate). The specificity is defined as the proportion of correctly identified negative events (True negative rate).

The limit of detection was determined in duplicates using serial dilutions of DNA from a wild-type GIST and from mutated GISTs with ten different mutations (p.V654A, p.T670A, p.T670K, p.N680K, p.D820Y, p.D820G, p.D820E, p.N822Y, p.D822K, p.Y823D, all from FFPE). The mean allele frequencies of the mutated GISTs used for the serial dilutions were calculated by independent, massively parallel sequencing runs for each assay. Mutated DNA was diluted to a concentration of 10 ng/μl and 10% allele frequency for each mutation respectively. The limit of detection was estimated as the point where the mutated sample could still be distinguished from a wild-type sample, before which the serial dilution reached a constant level (background noise).

## Results

### Primary mutations of the 33 primary GISTs

Previously determined *KIT* exon 9 and exon 11 mutations were verified in 29 of the 33 primary GISTs using the GS Junior (Roche). After repeating the experiment, three samples were still not evaluable and showed no coverage for exon 9 or 11 due to a low DNA content or highly fragmented DNA. However, two of these three samples were evaluable for exon 13, 14 and 17. All three samples could be investigated with the MiSeq™ (Illumina) assays. Thus, they were not excluded from this study. One sample showed a wild-type sequence in exon 11 instead of the p.V559G mutation. Using the Qiagen panel on the MiSeq™ (Illumina), the mutational status of *KIT* exon 9 and 11 of all 33 primary GISTs was confirmed (Table 1). Differences in the nomenclature of sequence variants were seen but could be resolved by renaming the mutations according to the recent HGVS nomenclature of gene variations [22]. The allele frequencies of exon 9 and 11 mutations in the primary GISTs varied between the GS Junior (Roche) and the MiSeq™ analysis. A difference of 1.8 – 91.4% was seen in samples between these two platforms (Additional file 4).

### Assay sensitivity, specificity and limit of detection

The sensitivity and specificity of each assay is shown in Table 3. For validation of the MiSeq™ (Illumina) assay the 36 secondary GIST samples were used. For validation of the GS Junior (Roche) a different set of 18 samples with known mutational status was used. The sensitivity and specificity of the GS Junior (Roche) and the Qiagen panel on the MiSeq™ (Illumina) was 100%. The sensitivity of the AmpliSeq panel on the MiSeq™ (Illumina) was only 93%. Using this panel, four of the exon 11 mutations (p.M552_K558del, p.M552_V559del, K550_K558del, c.1648-5_1672del) could not be detected as these mutations were at the amplicon boundaries and primer binding sites. The specificity of the AmpliSeq panel was 100% (Table 3). Thus, all secondary *KIT* mutations could be detected with all three assays.

**Table 3:** **Validation of the three assays used**

Assay	Sensitivity	Specificity	Limit of detection
GS Junior	100% (21/21)	100% (69/69)	1%^#^
MiSeq™ - Qiagen panel	100% (77/77)	100% (118/118)	0.03 – 0.25%^#^
MiSeq™ - AmpliSeq panel	93% (69/74)	100% (83/83)	0.02 – 0.45%^#^

Shown are the sensitivity, specificity and limit of detection for each assay.

Sensitivity: Proportion of correctly identified positive events (True positive rate).

Specificity: Proportion of correctly identified negative events (True negative rate).

(/): (number of detected/number of expected events).

^#^See Additional file 5 for detailed information.

The limit of detection determines the lowest detectable amount of mutated alleles in a background of wild-type DNA. In this study, the limit of detection was determined for ten different secondary *KIT* mutations. The limit of detection for each mutation tested was 1% on the GS Junior (Roche). For the MiSeq™ (Illumina) the limit of detection differed depending on the position of the secondary mutation (Table 3, Additional file 5) It spread from 0.03 – 0.25% on the MiSeq™ (Illumina) with the Qiagen panel and 0.02 – 0.45% with the AmpliSeq panel. Exemplarily, two of the serial dilutions illustrating the limit of detection, including the coverage and allele frequencies for each dilution step, are shown in Additional file 6.

### Performance of the GS Junior (Roche) pyrosequencing and the GeneRead Mix-n-Match DNAseq Gene Panel (Qiagen) on the MiSeq™ (Illumina)

The GS Junior (Roche) runs yielded in 78,200 – 116,710 passed filter reads and the MiSeq™ (Illumina) runs with the Qiagen panel yielded in 19.89 – 23.04 million passed filter reads, showing an increase in sequencing depth of around 200-fold. The quality of all GS Junior (Roche) and MiSeq™ (Illumina) runs were in the upper range for massively parallel sequencing according to manufacturer’s specifications.

The aligned sequencing reads per sample (four amplicons) were 4,424 – 29,584 on the GS Junior (Roche) and the mean coverage per sample (78 amplicons) were 450,879 – 5,551,341x on the MiSeq™ (Illumina) with the Qiagen panel.

### Analysis of secondary mutations in the 33 primary GISTs

**Figure 2:**
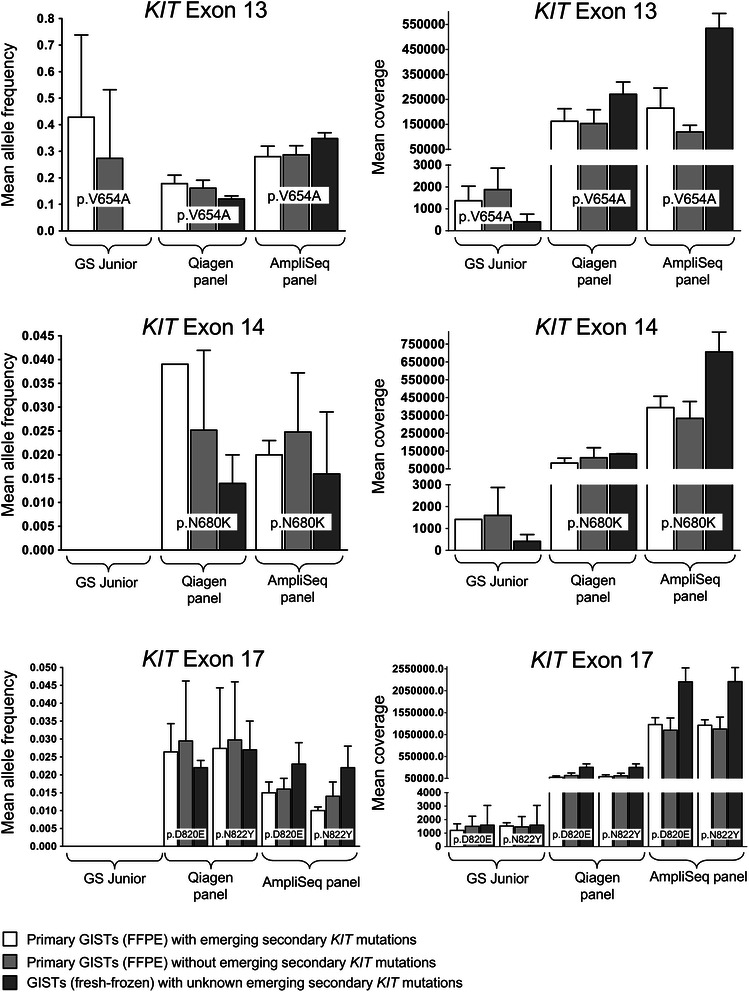
Analysis of minor variants of secondary *KIT* mutations in GISTs prior to imatinib therapy. Shown are the mean allele frequency (± the standard deviation) and mean coverage (± the standard deviation) at the positions of the mutations p.V654A (exon 13), p.N680K (exon 14), p.D820E (exon 17) and p.N822Y (exon 17). At each mutation position the results are shown for each of the three panels used: the GS Junior panel, the Qiagen panel and the AmpliSeq panel. The different coloured graphs illustrate the results of primary GISTs (FFPE) with (white) and without (grey) emerging secondary *KIT* mutations in the recurrent tumours and of GISTs (fresh-frozen) with unknown emerging secondary *KIT* mutations (dark grey). All measured allele frequencies are below the determined limit of detection (see corresponding Table 4).

**Figure 3:**
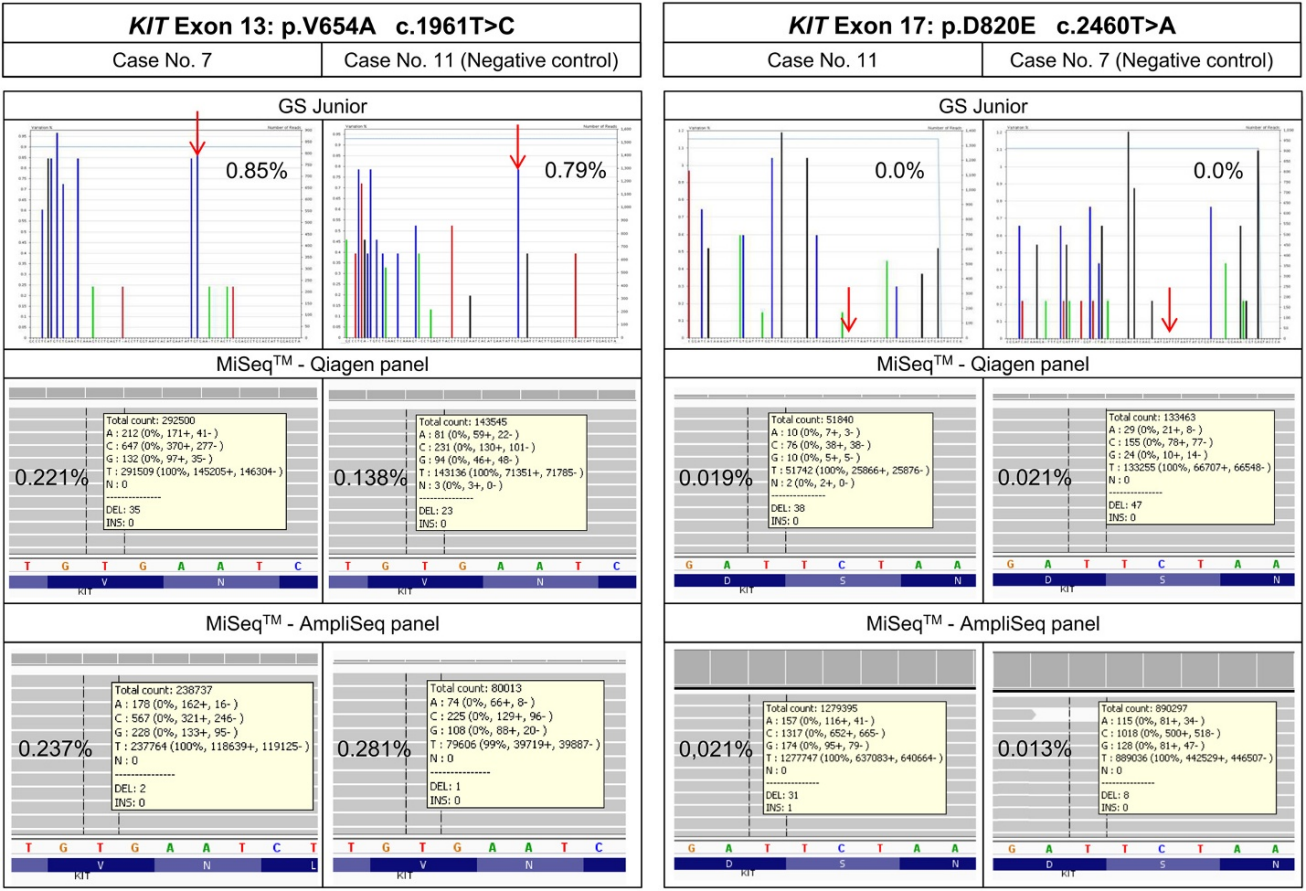
Results of minor variants of secondary *KIT* mutations of case 7 and 11 prior to therapy. Mean allele frequency of p.V654A and p.D820E substitutions for cases with and without emerging *KIT* exon 13 and exon 17 mutations determined by GS Junior (Roche) and MiSeq™ (Illumina) sequencing. The arrow indicates the position of the substitution.

The massively parallel sequencing results for the 33 primary GISTs were checked for the corresponding emerging secondary mutations that occurred in the lesions. In the 11 primary tumour samples with secondary *KIT* exon 13 mutation (c.1961 T > C, p.V654A) in the recurrent tumour, minor percentages were seen with the GS Junior (Roche). However, when analysing the remaining primary GISTs of the FFPE collective without later emerging secondary p.V654A resistance mutations as a negative control, the substitution was detected with the same mean allele frequency and were considered background noise (Table 4, Figure 2, Figure 3, Additional file 4). On the GS Junior (Roche) minor allele frequencies were observed only at the position of the secondary mutation p.V654A. No mutated alleles were detected with the GS Junior (Roche) at all other positions of known secondary mutations.

**Table 4:** **Summary of allele frequencies at each secondary** ***KIT*** **mutation position in primary GISTs**

Mutation	Assay	With emerging secondary mutation [%]	Without emerging secondary mutation [%]	Limit of detection [%]
p.V654A	GS Junior	0.000 - 0.850	0.000 - 0.790	1.00
	Qiagen panel	0.146 - 0.248	0.107 - 0.233	0.25
	AmpliSeq panel	0.216 - 0.415	0.254 - 0.363	0.45
p.N680K	GS Junior	0.000	0.000	1.00
	Qiagen panel	0.039	0.006 - 0.092	0.10
	AmpliSeq panel	0.013 - 0.026	0.008 - 0.055 (0.385)	0.07
p.D820E	GS Junior	0.000	0.000	1.00
	Qiagen panel	0.019 - 0.035	0.010 - 0.094	0.10
	AmpliSeq panel	0.010 - 0.021	0.012 - 0.028	0.03
p.N822Y	GS Junior	0.000	0.000	1.00
	Qiagen panel	0.017 - 0.047	0.011 - 0.074	0.08
	AmpliSeq panel	0.008 - 0.012	0.008 - 0.019	0.02

Shown are the allele frequencies in primary GISTs (FFPE) with and without emerging secondary mutation in the recurrent tumours in comparison to the limit of detection. The positions of the mutations p.V654A, p.N680K, p.D820E, p.N822Y were analysed with each assay.

[%]: Allele frequency in percent.

(): Falsely higher allele frequency due to read bias in one run.

To increase the sequencing depth and to decrease amplification artefacts by sequencing only one sample, 12 identical libraries of the same case with the same barcode were loaded on the GS Junior (Roche). With this approach we were able to increase the coverage from 828 to 48,087x and decrease the background noise from 1 to 0.4%, while at the same time decreasing the allele frequency at the position of the p.V654A mutation from an allele frequency of 0.85 to 0.16% (Additional file 7A).

The higher sequencing depth of the MiSeq™ (Illumina) led to similar results. With the Qiagen panel the mean allele frequency of the p.V654A mutation was the same between primary GISTs with and without emerging p.V654A mutation. Minor mutated allele frequencies at the positions of secondary mutations in exon 14 and 17 of the *KIT* gene were not detected with the GS Junior (Roche). With the MiSeq™ (Illumina) mutated allele frequencies at these positions were detected at lower frequencies than for the p.V654A mutation, but again no difference could be seen between primary GISTs with and without later emerging secondary mutations and were again considered background noise (Table 4, Figure 2, Figure 3, Additional file 4).

When analysing only one same sample at different coverages with the Qiagen panel on the MiSeq™ (Illumina) instead of the GS Junior (Roche), the same effect could be observed; an increase in the sequencing depth decreased the background noise (Additional file 7B).

In the cases 30, 31, 32 and 33, secondary *KIT* mutations were identified with a high allele frequency (Additional file 4). After repeated examination of the clinical history of the primary tumours, these tumours turned out to be progressed lesions under therapy. Due to insufficient clinical data the tumours were initially identified as primary tumours with activating *KIT* exon 11 mutations and no secondary resistance mutations were evaluated.

### Tumour segmentation into subregions and performance of the Ion AmpliSeq™ Custom DNA Panel (Life Technologies) on the MiSeq™ (Illumina)

**Figure 4:**
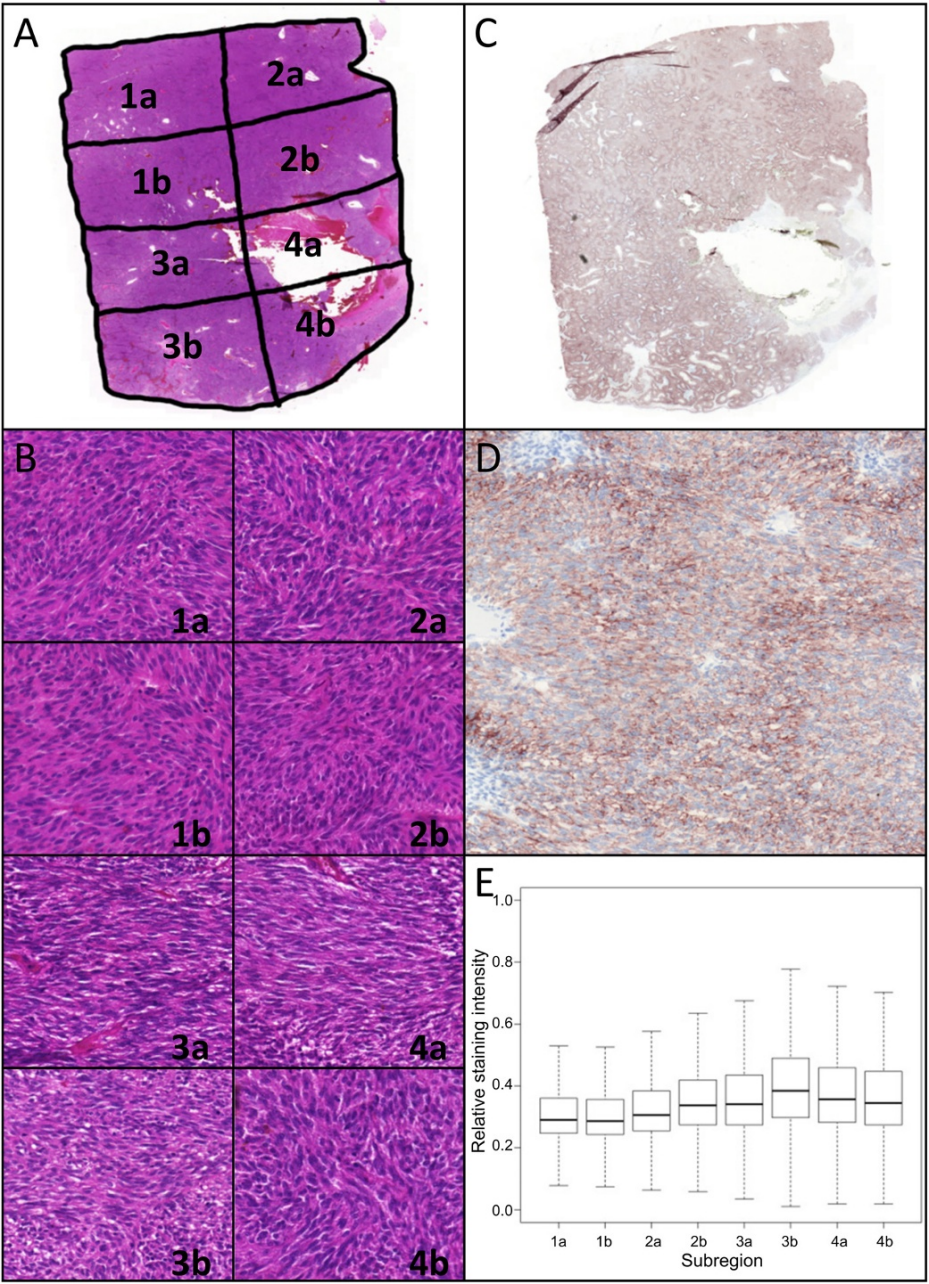
Histological characteristics of subregions of case 7. **(A)** Overview of segmented H&E stain (magnification 10x). **(B)** H&E stain of each subregion (magnification 200x). **(C)** Overview of CD117 stain (magnification 10x). **(D)** 200x magnification of subregion 2b. **(E)** Quantitative immunohistochemistry. Image analysis of 1.85 mm^2^ per subregions. Shown are the median, the 95% confidence interval and the standard deviation.

Five of the primary GISTs were segmented into a total of 52 equal subregions in order to increase the sensitivity, the sequencing depth and the likelihood of detecting a minor resistant subclone by decreasing the wild-type background,. The five selected primary GISTs showed different primary mutations in *KIT* exon 11 and different emerging secondary *KIT* mutations in exon 13, 14 and 17. Additionally, these samples were large resections of different localisations with sufficient tumour material for segmentation. The subregions showed differences neither in morphology nor immunohistochemical staining pattern and intensity (Figure 4). By quantitative immunohistochemistry of the CD117 staining no categorical differences were noticed.

The MiSeq™ (Illumina) runs of the 52 subregions with the AmpliSeq panel yielded 15.38 – 19.66 million passed filter reads. The quality of the runs was in concordance with the manufacturer’s specifications. The mean coverage per sample (six amplicons) was between 437,619 and 3,046,805x. For all 52 samples the allele frequency at the position of the *KIT* substitutions exon 13 p.V654A, exon 14 p.N680K, exon 17 p.D820E and exon 17 p.N822Y was determined. For each substitution the same minor allele frequency could be detected in primary tumours with and without the corresponding emerging secondary resistance mutation. Even an increase in the sequencing depth with a coverage of 1.574 Million in exon 17 did not lead to different results. For exon 13 and 17 the allele frequency was even higher in the negative control samples (Table 4, Figure 2).

In one run with 15 negative control subregions, the forward and the reverse strand of the exon 14 substitution p.N680K showed an imbalance in sequence reads, which led to a false higher allele frequency (Additional file 8). When excluding these 15 subregions the mean mutated allele frequency was reduced to the same frequency as the other negative control samples. As the imbalance was only seen in one run with negative control subregions, the detection of minor subclones was not affected. Exemplarily, the results of two cases for all three assays are shown in Figure 3.

### Comparison of assay performance in DNA extracted from fresh-frozen and FFPE tissue

For the fresh-frozen samples the mutational status after therapy was not known. Therefore, the same four most common secondary *KIT* mutations, as described above, were analysed.

With the GS Junior (Roche) six fresh-frozen samples were analysed and no mutated allele frequencies at the positions of secondary mutations were detected. With the MiSeq™ (Illumina) three fresh-frozen samples were analysed and minor frequencies of the mutated allele could be detected. However, the allele frequencies were in the same range as in the analysed primary FFPE samples and were determined to be background noise (Figure 3, Additional files 8, 9 and 10).

## Discussion

The development of secondary resistance mutations during imatinib therapy is the most common resistance mechanism in GISTs. Experimental evidence of whether secondary mutations are pre-existing in minor subclones or develop “de novo” during therapy has yet to be provided and would help to develop new therapeutic strategies in GISTs.

In this study, 33 primary GISTs with known progressed disease and secondary resistance mutations were analysed on the GS Junior (Roche) and on the MiSeq™ (Illumina) with three different assays.

With an achieved sensitivity of 0.02% mutated alleles in the background of wild-type alleles for *KIT* exon 17 p.N822Y, p.N822K and p.Y823D mutations on the MiSeq™ (Illumina) with the AmpliSeq panel, no pre-existing subclones were detected with any of the three assays. The limit of detection varied between individual secondary mutations. Additionally, it could be seen that at each position of secondary mutations some negative samples (samples without later emerging secondary mutations) had higher allele frequencies than the samples with later emerging secondary mutations. Thus, the threshold used to distinguish positive from negative cases was determined for each position of secondary mutations by the allele frequencies of the negative samples, correlating with the limit of detection.

On both systems the sensitivity of the assay was limited by background noise. Particularly high background noise and artificial T > C substitutions at the position of the p.V654A mutation posed a problem and led to a higher detection limit. Artificial T > C transitions could be artefacts which are associated with formalin fixation and are a common problem in FFPE material, especially when using small biopsies and low DNA content [23,24]. Formalin cross-links cytosine nucleotides on either strand and/or deaminates cytosine to uracil and adenine to hypoxanthine. During PCR reaction the Taq polymerase incorporates an adenine instead of a guanine and a cytosine instead of a thymine and non-reproducible C<>T and G<>A mutations are created [24-26].

Forshew et al. showed in 47 FFPE samples that background frequencies of artificial substitutions were around 0.1% and varied depending on base substitution and loci [27].

To reduce the effect of fixation artefacts and background noise three approaches were chosen: the sequencing depth was increased, fresh-frozen material was analysed and FFPE material was treated with uracil-N-glycosylase (UDG).

It is common knowledge that the detection of low mutated allele frequencies depends among others on the sequencing depth. Thus, an increase in the sequence coverage leads to an increase in the detection sensitivity of somatic variants by decreasing the background noise [28-32]. This effect was also seen in our study. However, in our study a much higher increase in the sequencing depth was achieved, which has not been published yet. In our study, this approach was first shown on the GS Junior (Roche). We increased the sequencing depth, and thus the method sensitivity, by sequencing 12 independent libraries with the same barcode of only one case on the GS Junior (Roche). By this approach, we not only increased the method sensitivity by increasing the sequencing depth, we also decreased amplification errors and thus the background noise by combining 12 independent PCR reactions. Here, we were able to increase the coverage from 828 to 48,087 and decrease the background noise from 1 to 0.4%, while at the same time decreasing the allele frequency at the position of the p.V654A mutation from an allele frequency of 0.85 to 0.16%. On the MiSeq™ (Illumina) we could observe the same effect of coverage increase and background noise decrease, when analysing the same sample at different coverages. Here, we used one PCR reaction per sample only.

Generally speaking, with the MiSeq an approximately 70-fold increase in sequencing depth led to an at least 3-fold decrease in the background noise. However, the principle described above could not be observed in all experiments. On the MiSeq™ (Illumina), the AmpliSeq panel showed in some amplicons a more than 10-fold increase in the sequencing depth in comparison to the Qiagen panel but a reduction of the background noise at the positions of the secondary mutations could not be observed an each position.

Thus, in our study, the reduction of background noise and increase in detection sensitivity by increasing the sequencing depth of the method led to the same results. No pre-existing secondary mutation exceeded the background noise (the allele frequency at the relevant position of the secondary mutations) in the primary tumour samples.

We analysed six fresh-frozen samples with the GS Junior (Roche) and three fresh-frozen samples with both MiSeq™ (Illumina) panels. With the GS Junior (Roche) no minor frequencies of mutated alleles were seen at four positions of secondary mutations (p.V654A, p.N680K, p.D820E, p.N822Y). With the MiSeq™ (Illumina) minor allele frequencies of the mutated allele were detected, but the frequencies and the sensitivity were the same as with the FFPE material and were thus determined as background noise.

Spencer et al. showed that most high-quality base discrepancies were not significantly different between FFPE und fresh-frozen material, and are rather due to sequencing errors and DNA damage. Only C > T and G > A transitions were significantly increased when comparing FFPE and fresh-frozen material [33].

Nguyen et al. showed that transitions are especially prone to sequencing errors due to base-pairing and reading errors. They showed >1% erroneous sequences independent of the material source [34]. Another study showed the presence of 0.05 – 1% sequencing errors with human cells and bacterial DNA [35].

Additionally, 19 of the 33 primary GISTs were extracted with the GeneRead DNA FFPE KIT (Qiagen) and sequenced with the AmpliSeq panel. This kit uses UDG, which reduces C > T (and G > A) sequence artefacts [26,36]. Do et al. showed that UDG treatment reduces the allele frequency of G > A artefacts from 0.1 to 2.07% to 0.1 to 0.7%. However, as UDG removes uracil from damaged FFPE DNA only C > T and respectively G > A transitions are reduced. Therefore no reduction in T > C artefacts at the p.V654A position was seen.

At the positions of exon 14 and exon 17 substitutions the allele frequencies of the mutated allele and respectively the background noise were often as low as 0.02% on the MiSeq™ (Illumina). These substitutions were mostly transversions G<>T and T<>A, which are not affected by fixation artefacts or sequencing errors. Nevertheless, no minor resistant subclones could be detected at these positions.

Further, low-diversity libraries, i.e. libraries with only a few amplicons, may lead to an imbalance in sequence reads of the forward and reverse strand in MiSeq™ (Illumina) runs with normal cluster densities. Due to the low number of different amplicons, the likelihood of clusters of the same amplicon appearing next to each other on a flow cell is higher than in MiSeq™ (Illumina) runs with more diverse libraries. When analysing low-diversity libraries, the MiSeq™ (Illumina) cannot distinguish between the individual clusters and might detect the wrong nucleotide. As this reading error occurs in the two sequencing runs independently, it results in an imbalance between the two sequence reads and leads to the detection of false positives with a falsely higher allele frequency. To increase the run quality, it is stated that the cluster density should be decreased and that only balanced sequence reads should be analysed [37-39]. This approach was also applied in this study. To show the risk of imbalanced sequencing reads and false positives when using low-diversity libraries, one run showing imbalanced sequencing reads at the position of the secondary mutation in KIT exon 14 (p.N680K) was included in this paper. In this run, only cases without later emerging p.N680K mutation were included.

In addition to the massively parallel sequencing, a wild-type blocking LNA-mediated clamping assay (TIB Molbiol) for the p.V654A substitution was used in this study. With a sensitivity of 0.4% the assay yielded no other results than the massively parallel sequencing (data not shown). All samples were wild-type for p.V654A.

New large-scale sequencing approaches have revealed the extensive intra- and intertumour heterogeneity in many cancers [40-42]. In renal cancer 63 – 69% of mutations were not detectable in every tumour region [40]. Therefore, the detection of subclonal mutations is important as these subclones may contribute to primary and acquired resistance [43-45].

This tumour heterogeneity and the development of polyclonal resistance mutations during therapy has also been described for GISTs [5,10,11]. Wardelmann et al. showed that a biopsy is not representative for the whole tumour [5,11]. In our study, five of the 33 primary GISTs were segmented into a total of 52 subregions to minimise the analysed tumour region and reduce the wild-type background. However, this approach led to similar results and no minor resistant subclones could be detected prior to tyrosine kinase inhibitor therapy. It remains unresolved whether the detection limit of two mutated clones in 10,000 wild-type clones was not high enough, whether heterogeneous tissue samples are, per se, not suitable for the detection of very small subpopulations of mutated cells or whether in general no subclones were present.

The assessment of the probability of pre-existing resistant subclones is an ongoing challenge. In some tumour entities, pre-existing resistant subclones could be detected. In colorectal carcinoma *KRAS* resistance mutations were detected with an allele frequency of 0.2%. In non-small cell lung cancer p.T790M *EGFR* resistance mutations were detected with an allele frequency of 0.4 – 0.02% [4,7,8]. These mutations were mainly detected with TaqMan assays, massively parallel sequencing approaches and mathematical modelling. The method sensitivity in our study was within the same range. However, in our study no pre-existing resistant subclones were detected. This is in concordance with published theories, which state that in GIST resistance mutations develop “de novo” during therapy as GIST patients with developing secondary resistance mutations are commonly treated longer with the tyrosine kinase inhibitor imatinib than resistant patients without these mutations [14]. Hence, it is assumed that clonal selection of pre-existing resistance mutations in GIST is unlikely.

In the previous lung and colorectal carcinoma studies, mentioned above, pre-existing subclones were determined in blood samples and cell cultures.

Therefore, the analysis of circulating tumour DNA may be promising in the early detection of resistance mutations, which will overcome tissue heterogeneity and formalin fixation, and may also be useful in the detection of pre-existing resistant subclones [46-48].

Further, mathematical models have already been used and might be useful to predict pre-existing resistant minor subclones in combination with experimental and clinical data in GISTs [15].

## Conclusion

Despite the use of ultrasensitive methods available nowadays and a minimal sensitivity level of 0.02% varying between individual secondary mutations, this study detected no pre-existing resistant subclones. This result is based on the analysis of 33 primary FFPE GISTs with known secondary resistance mutation and nine fresh-frozen GISTs prior to therapy.

Our results support the theory that such mutations develop under tyrosine kinase inhibitor treatment by “de-novo” mutagenesis in GISTs. On the other hand, either the methods employed might still not be sensitive enough or heterogeneous tissue samples, per se, might not be suitable for the detection of very small subpopulations of mutated cells.

## Additional files

Additional file 1:
**Target specific primers for GS Junior library preparation (FFPE).**

Additional file 2:
**Target specific primers for GS Junior library preparation (fresh-frozen).**

Additional file 3:
**Primers of the Ion AmpliSeq™ Custom DNA Panel (Life Technologies) for the KIT gene (FFPE/fresh-frozen).**

Additional file 4:
**Results of the 33 sequenced primary tumours with the GS Junior (Roche) and the GeneRead Mix-n-Match DNAseq Gene Panel (Qiagen) on the MiSeq™ (Illumina).**

Additional file 5:
**Limit of detection for each assay on the GS Junior (Roche) and on the MiSeq™ (Illumina) for nine different secondary mutations.**

Additional file 6:
**Determination of the limit of detection for the mutation p.V654A with the GeneRead Mix-n-Match DNAseq Gene Panel (Qiagen) and Ion AmpliSeq™ Custom DNA Panel (Life Technologies) on the MiSeq™ (Illumina).**

Additional file 7: **Illustration of the reduction of background noise by increasing the sequencing depth of the same case on the GS Junior (Roche) (A) and on the MiSeq™ (Illumina) with the GeneRead Mix-n-Match DNAseq Gene Panel (Qiagen)(B) at the position of the mutation p.V654A.** The red arrow indicates the position of the p.V654A mutation. On the GS Junior (Roche) amplification artefacts and thus background noise were reduced additionally by combining 12 independent PCR reactions of the same case.
Additional file 8:
**Results of the 52 sequenced subregions with the Ion AmpliSeq™ Custom DNA Panel (Life Technologies) on the MiSeq™ (Illumina).**

Additional file 9:
**Results of the 6 sequenced fresh-frozen primary tumours with the GS Junior (Roche).**

Additional file 10
**Results of the 3 sequenced fresh-frozen primary tumours with the GeneRead Mix-n-Match DNAseq Gene Panel (Qiagen) and the Ion AmpliSeq™ Custom DNA Panel (Life Technologies) on the MiSeq™ (Illumina).**

## Abbreviations

GIST: Gastrointestinal stromal tumour
FFPE: Formalin-fixed, paraffin embedded
H&E: Haematoxylin-eosin
Qiagen panel: GeneRead Mix-n-Match DNAseq Gene Panel (Qiagen)
AmpliSeq panel: Ion AmpliSeq^TM^ Custom DNA Panel (Life Technologies)
AVA: Amplicon Variant Analyser
IGV: Integrative Genomics Viewer
UDG: Uracil-N-glycosylase
