# Supplementary figures and images for: Massively parallel sequencing fails to detect minor resistant subclones in tissue samples prior to tyrosine kinase inhibitor therapy
Source: BMC Cancer. 2015 Apr 15;15:291. doi: 10.1186/s12885-015-1311-0 (PMC4404105; doi:10.1186/s12885-015-1311-0)

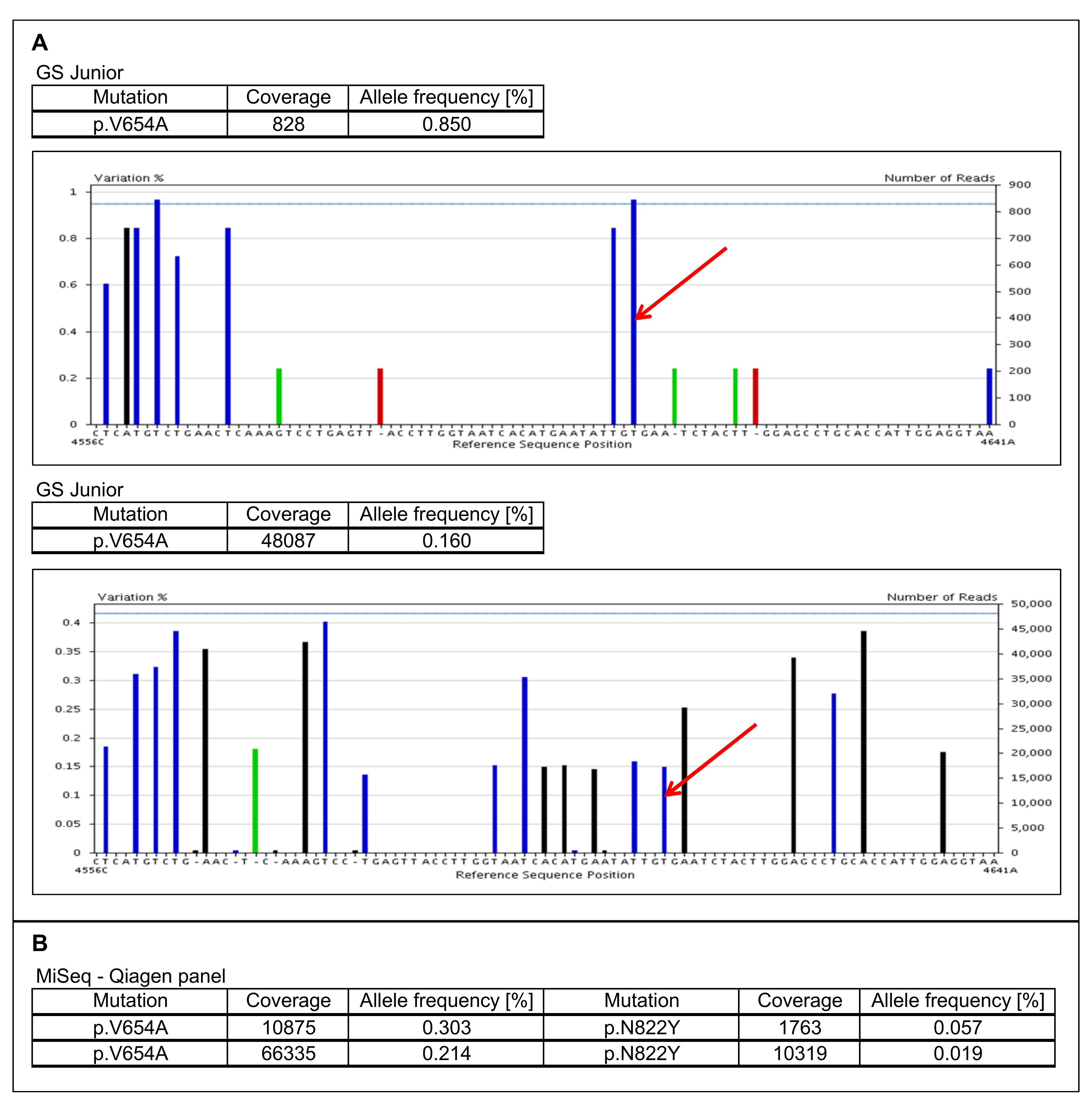

Supplement: Additional file 7: — Illustration of the reduction of background noise by increasing the sequencing depth of the same case on the GS Junior (Roche) (A) and on the MiSeq™ (Illumina) with the GeneRead Mix-n-Match DNAseq Gene Panel (Qiagen)(B) at the position of the mutation p.V654A. The red arrow indicates the position of the p.V654A mutation. On the GS Junior (Roche) amplification artefacts and thus background noise were reduced additionally by combining 12 independent PCR reactions of the same case. [file 12885_2015_1311_MOESM7_ESM.tiff]
